# Prognostic Value of the Metastatic Lymph Node Ratio in Patients With Resectable Carcinoma of Ampulla of Vater

**DOI:** 10.1097/MD.0000000000001859

**Published:** 2015-10-23

**Authors:** Chih-Ho Hsu, Tai-Di Chen, Chun-Yi Tsai, Jun-Te Hsu, Chun-Nan Yeh, Yi-Yin Jan, Ta-Sen Yeh, Wen-Chi Chou, Keng-Hao Liu

**Affiliations:** From the Department of General Surgery, Chang Gung Memorial Hospital, Linkou, Taiwan (C-HH, C-YT, J-TH, C-NY, Y-YJ, T-SY, K-HL), Department of Anatomical Pathology, Chang Gung Memorial Hospital, Linkou, Taiwan (T-DC), Graduate Institute of Clinical Medical Sciences, College of Medicine, Chang Gung University, Taiwan (T-SY, W-CC); and Department of Hematology and Oncology, Chang Gung Memorial Hospital, Linkou, Taiwan (W-CC).

## Abstract

Patients with carcinoma of the ampulla of Vater (CAV) have better outcomes among periampullary malignancies. However, little is known about the metastatic lymph node ratio (LNR) as a prognostic factor for resectable CAV. We retrospectively reviewed our CAV patients undergoing curative surgery and analyzed their prognostic factors.

A total of 212 CAV patients who received radical surgery at Chang Gung Memorial Hospital, Linkou, between 2000 and 2010 were admitted in this study. The lymph node ratio was defined as the number of metastatic lymph nodes (LNs) divided by the total number of LNs removed. The patients’ demographic data, comorbidities, operation type, and tumor features were analyzed retrospectively for survival prediction of patients.

The median age of the patients was 62 years, and 57% of the patients were men. The surgical procedure was standard pancreaticoduodenectomy and pylorus-preserving pancreaticoduodenectomy in 53% and 47% of the patients, respectively. The median follow-up duration was 32.6 months, and 50% of the patients had died by the end of the study. The median overall survival time (OS) and disease-free survival time (DFS) were 65.8 and 33.7 months, respectively. In multivariate analysis, patients with a metastatic LNR >0.056 had a significantly poor prognosis in both OS and DFS.

A metastatic LNR >0.056 predicted a poor DFS and OS in CAV patients after radical surgery. Greater awareness on the impact of metastatic LNR may help clinicians provide appropriate adjuvant treatment for high-risk CAV patients.

## INTRODUCTION

Carcinoma of the ampulla of Vater (CAV) is defined as a malignancy involving the papilla of Vater, a complex region composed of 3 distinct anatomical structures: the common bile duct, the pancreatic duct, and the duodenum. Although CAV is a relatively uncommon neoplasm, the incidence of CAV is ∼4 to 4.8 cases per million population per year.^1^ Carcinoma of the ampulla of Vater is the one of the most common periampullary malignancies for which patients receive pancreaticoduodenectomy (PD) and pylorus-preserving pancreaticoduodenectomy (PPPD).^2^ Compared to patients with pancreatic adenocarcinoma, patients with CAV have a better outcome that may contribute to an earlier appearance of obstructive symptoms and more favorable tumor behavior.^3^ Many negative prognostic factors, including positive resection margins, larger tumor size, lymph node involvement, histological differentiation, and perineural and lymphatic invasion, have been well documented for resectable CAV.^4–8^ Recently, the metastatic lymph node number (LNN) and the ratio of metastatic lymph nodes to total resected lymph nodes (LNR) in CAV have also been investigated.^9–14^ Hurtuk et al were the first to note that a higher LNR was significantly associated with a poor outcome in 75 CAV patients after surgery.^10^ However, other studies have reported that LNRs were insignificant in multivariate analyses after adjusting for variables such as metastatic LNNs.^11,12,14^

The significance and optimal cutoff value of LNR with its relevance to prognosis in CAV is uncertain. This study aimed to assess the prognostic value of LNR in CAV patients following resection and to identify the optimal cutoff value of LNR in relation to prognosis.

## PATIENTS AND METHODS

### Patient Selection

A total of 332 patients diagnosed with CAV at Chang Gung Memorial Hospital, Linkou, between 2000 and 2010 were consecutively admitted in this retrospective cohort study. The following patients were excluded from survival analysis: (a) patients with major vascular encasement (superior mesenteric vein, superior mesenteric artery, or portal vein) or a T4 tumor resulting in macroscopic incomplete resection (N = 28); (b) patients diagnosed with distant metastasis with or without palliative surgical treatment (N = 58); (c) pathologic cell types that were neither adenocarcinoma nor poorly differentiated carcinoma (N = 10); (d) patients undergoing ampullectomy only (N = 11); (e) patients who died after the operation during admission, contributing to in-hospital mortality (N = 17). Finally, this study included 212 patients undergoing curative surgery. The local Institutional Review Board of Chang Gung Memorial Hospital (104–1696B) approved this study.

### Data Collection

Data on patient demographics, pre-existing comorbidity, cancer cell histological differentiation, pathological characteristics of the tumor, surgical method, and tumor stage were collected by retrospectively reviewing the medical records. The Charlson comorbidity index (CCI) was calculated according to the patients’ pre-existing comorbidities.^15^ The surgical procedures included standard PD and PPPD, depending on the surgeons’ preference. Regional lymphadenectomy included dissection of the lymph nodes in the hepatoduodenal ligament, along the superior mesenteric vessels, and on the surface of the pancreas. The need for adjuvant treatment, including chemotherapy or/and radiotherapy, was determined by the surgeon in cases with poor prognostic factors (positive resection margin, lymphovascular invasion, perineural invasion, or lymph node metastases). The tumor stage was registered according to the seventh edition of the pathological tumor-node-metastasis (pTNM) staging system issued by the American Joint Committee on Cancer (AJCC).^16^ Metastatic LNR was defined as the number of metastatic lymph nodes (LNs) divided by the total number of LNs removed. The dates on tumor recurrence and death were obtained from our institutional cancer registration center. The disease-free survival (DFS) and overall survival (OS) times were determined from the time of surgery to the time of tumor recurrence and death. All of the included patients were followed-up on until date of death or June 30, 2014.

### Statistical Analysis

The basic demographic data are summarized as n (%) for categorical variables and as the median within a range for continuous variables. Survival time was calculated using the Kaplan–Meier method. Univariate and multivariate analyses of DFS and OS for patients of all clinical characteristics were performed using the log-rank test and the Cox proportional hazards model.

The characteristics significantly associated with DFS and OS as identified by univariate analysis were entered into multivariate analysis. To avoid the interaction of LNR and LNN in the multivariate model, LNR rather than LNN was included for analysis in the multivariate model based on a higher chi-square value of LNR than LNN in the univariate analysis of disease-free survival (64.48 vs 43.60) and overall survival (58.97 vs 38.13). The hazard ratios (HRs) were estimated using multivariate Cox regression.

We used classification and regression trees (CART) analysis to determine the cutoff values of LNN and LNR for DFS and OS. The CART analysis is a statistical technique based on the binary recursive partitioning method.^17^ The program selected the variables that provided the optimal cutoff value to split variables into two subgroups with most significance in the survival-time outcome. Then each subgroup was further dichotomized by one of these variables into smaller groups with difference in the survival-time outcome. The process of partitioning would stop either because a subgroup was homogeneous for the survival-time outcome or because the subgroup was too small to segregate further. Additionally, CART analysis is nonparametric and can manage both numerical and categorical variables. The CART analysis was performed using RPART library in R (R Development Core Team, 2010), and the other statistical analyses were performed using SPSS v13.0 (SPSS Inc, Chicago, IL). All statistical assessments were considered significant at *P* <0.05.

## RESULTS

### Demographic Data

The demographic data of the patients are summarized in Table 1. The median age of the patients was 61 years (range: 34–90 years), and 56.6% of the patients were men. The median CCI of the patients was 3 (range: 2–8). Of the patients, 112 (52.8%) patients underwent PD, and 100 (47.2%) patients underwent PPPD. Well, moderately, and poorly differentiated histological differentiations were noted in 51 (24.1%), 141 (66.5%), and 20 (9.4%) patients, respectively. Microscopic examination revealed that lymphovascular and perineural invasions were detected in 80 (37.7%) and 51 (24.1%) patients, respectively.

**TABLE 1:**
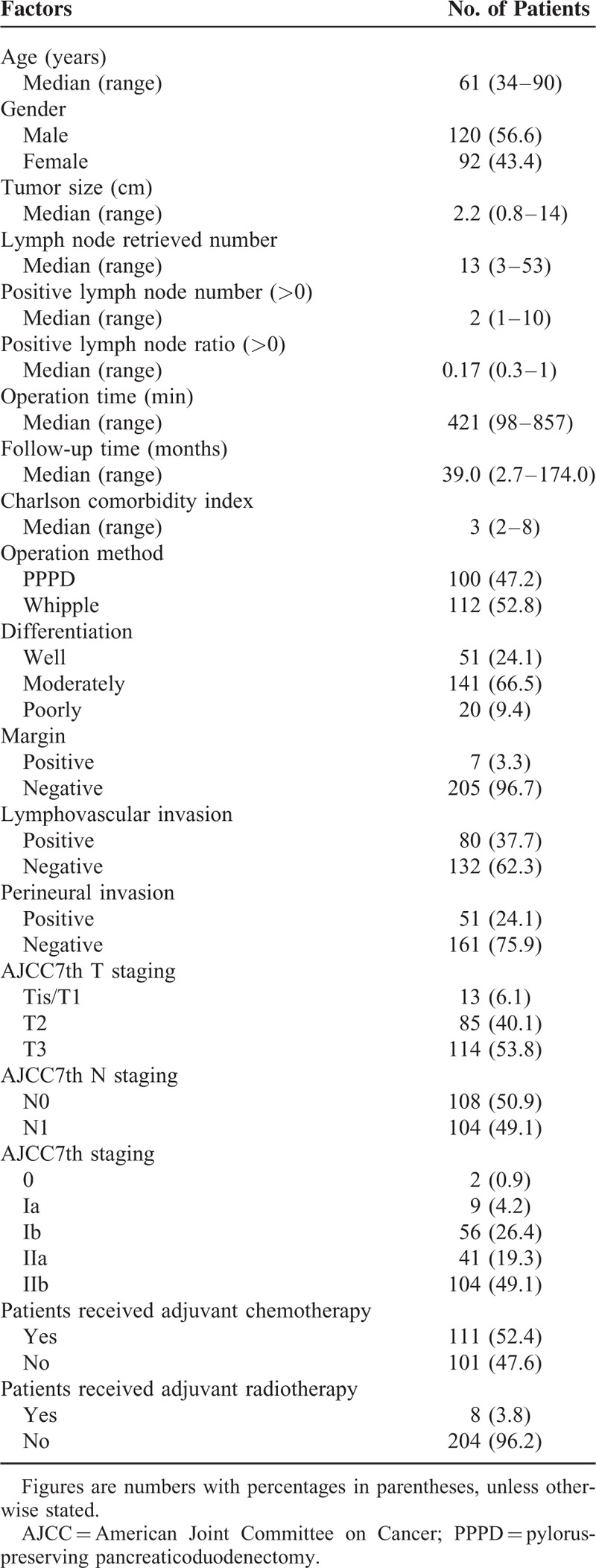
Clinicopathological Factors of Patients (N = 212)

The median diameter of the resected primary tumor was 2.2 cm (range: 0.8–14 cm). With regard to T stage, 6.1%, 40.1%, and 53.8% of patients were classified as having Tis/T1, T2, and T3 disease, respectively. The median lymph node retrieval number was 13 (range: 3–53), and lymph node metastases were noted in 104 (49.1%) patients. Based on the AJCC staging system (seventh edition), 2 (0.9%) patients had stage 0 diseases, 9 (4.2%) had stage IA disease, 56 (26.4%) had stage IB disease, 41 (19.3%) had stage IIA disease, and 104 (49.1%) had stage IIB disease. The median follow-up duration was 32.6 months (range: 0.1–174.0 months).

### Disease-Free Survival Analysis

Tumor recurrence was observed in 114 (51.4%) of 212 patients who could be evaluated for DFS. The median time to tumor recurrence was 33.7 months (95% confidence interval [CI] = 5.4–62.0). The 1-year, 3-year, and 5-year DFS rates were 71.6%, 48.8%, and 46.0%, respectively. The significant prognostic factors that influenced DFS in the univariate analysis were histological differentiation, AJCC 7th T staging and N staging, a metastatic LNR >0.056, lymphovascular invasion, and perineural invasion. No difference in the DFS was noted according to age, gender, comorbidities, surgical procedure, or microscopic resection margin. Only a metastatic LNR >0.056 (adjusted HR = 3.9; 95% CI = 2.4–6.3; *P* <0.001) was a significant prognostic factor in the multivariate analysis (Table 2).

**TABLE 2:**
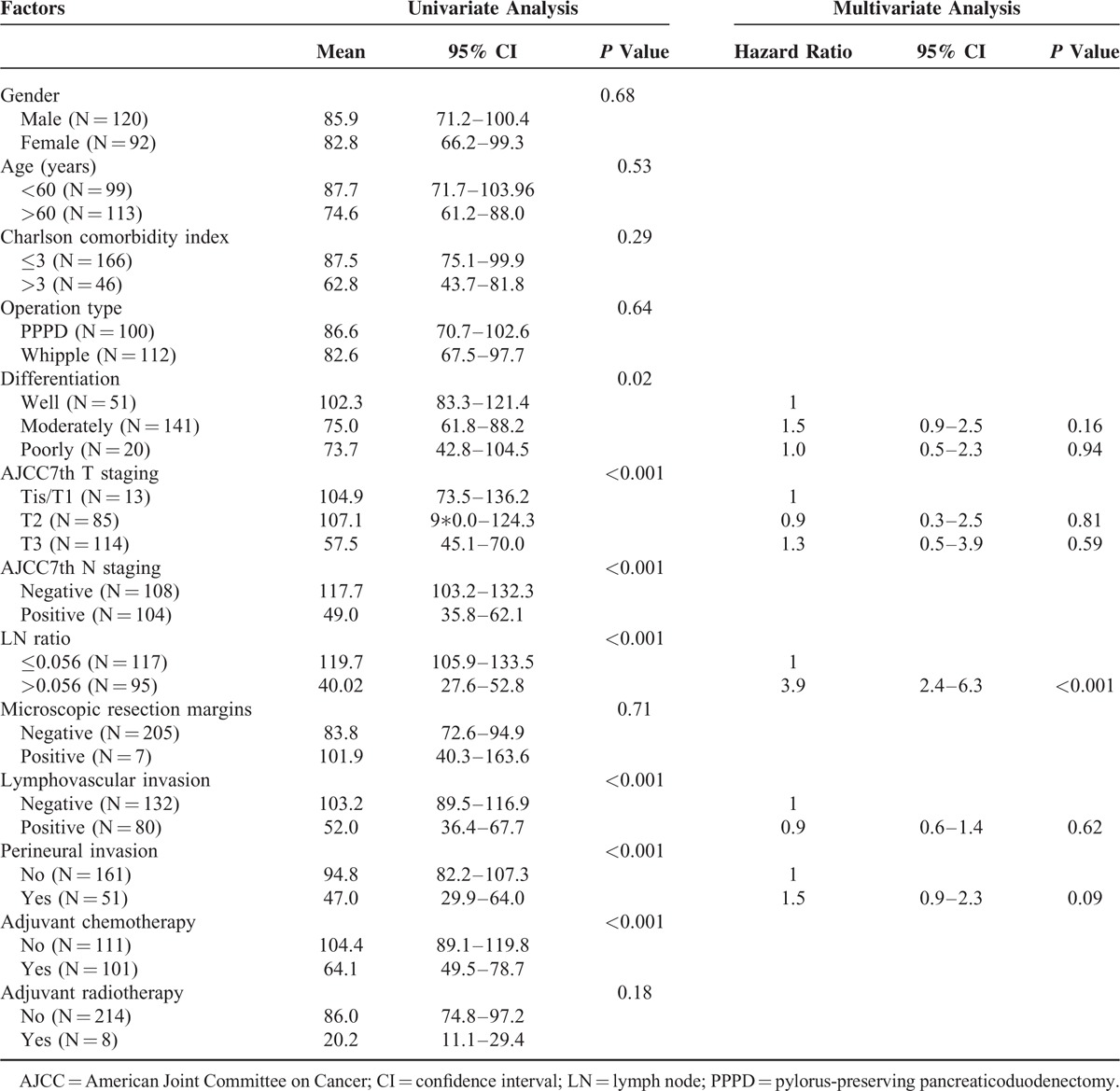
Univariate and Multivariate Analyses of Risk Factors Associated With Disease-Free Survival

**FIGURE 1:**
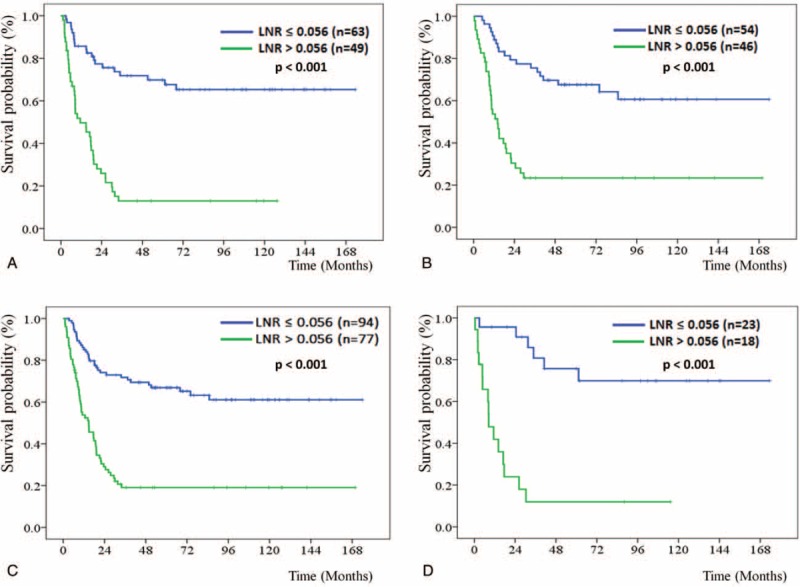
Kaplan–Meier disease-free survival curves for patients stratified with LNR ≤ 0.056 and >0.056 groups according to the operation method with pancreaticoduodenectomy (A) and pylorus-preserving pancreaticoduodenectomy, PPPD (B), as well as the total number of lymph node resections ≤ 20 (C) and >20 groups (D). LNR = lymph node ratio; PPPD = pylorus-preserving pancreaticoduodenectomy

Regarding the impact of LNR on DFS through different operation methods, patients who underwent PD or PPPD were further stratified by LNR ≤ 0.056 vs >0.056 for DFS analysis, respectively. The median DFS of the patients with LNR ≤ 0.056 and >0.056 did not reach 11.4 months (*P* <0.001) for patients who underwent PD (Fig. 1A) and did not reach 14.1 months (*P* <0.001) for patients who underwent PPPD (Fig. 1B), respectively. Based on the total number of lymph node resections, 80.6% and 19.4% of the patients were classified as lymph node resection number ≤ 20 and >20 groups, respectively. The median DFS of the patients with LNR ≤ 0.056 and >0.056 did not reach 14.8 months (*P* <0.001) for the patients with lymph node resection number ≤ 20 group (Fig. 1C) and did not reach 8.4 months (*P* <0.001) for the lymph node resection number >20 group (Fig. 1D), respectively.

### Overall Survival Analysis

During the follow-up period, 106 (50%) out of 212 patients died. The median OS time was 65.8 months (95% CI = 31.4–100.3), and the 1-year, 3-year, and 5-year OS rates were 87.5%, 57.3%, and 51.1%, respectively. Gender, age, CCI, operative procedure type, surgical complications, and status of resection margins were not related to OS. However, OS was statistically related to tumor differentiation, AJCC 7th T staging and N staging, a metastatic LNR of >0.056, lymphovascular invasion, and perineural invasion. In the multivariate analysis, metastatic LNR >0.056 (adjusted HR = 4.3; 95% CI = 2.6–7.1; *P* <0.001) and perineural invasion (adjusted HR = 1.7; 95% CI = 1.1–2.7; *P* = 0.02) were significantly related to poor OS (Table 3).

**TABLE 3:**
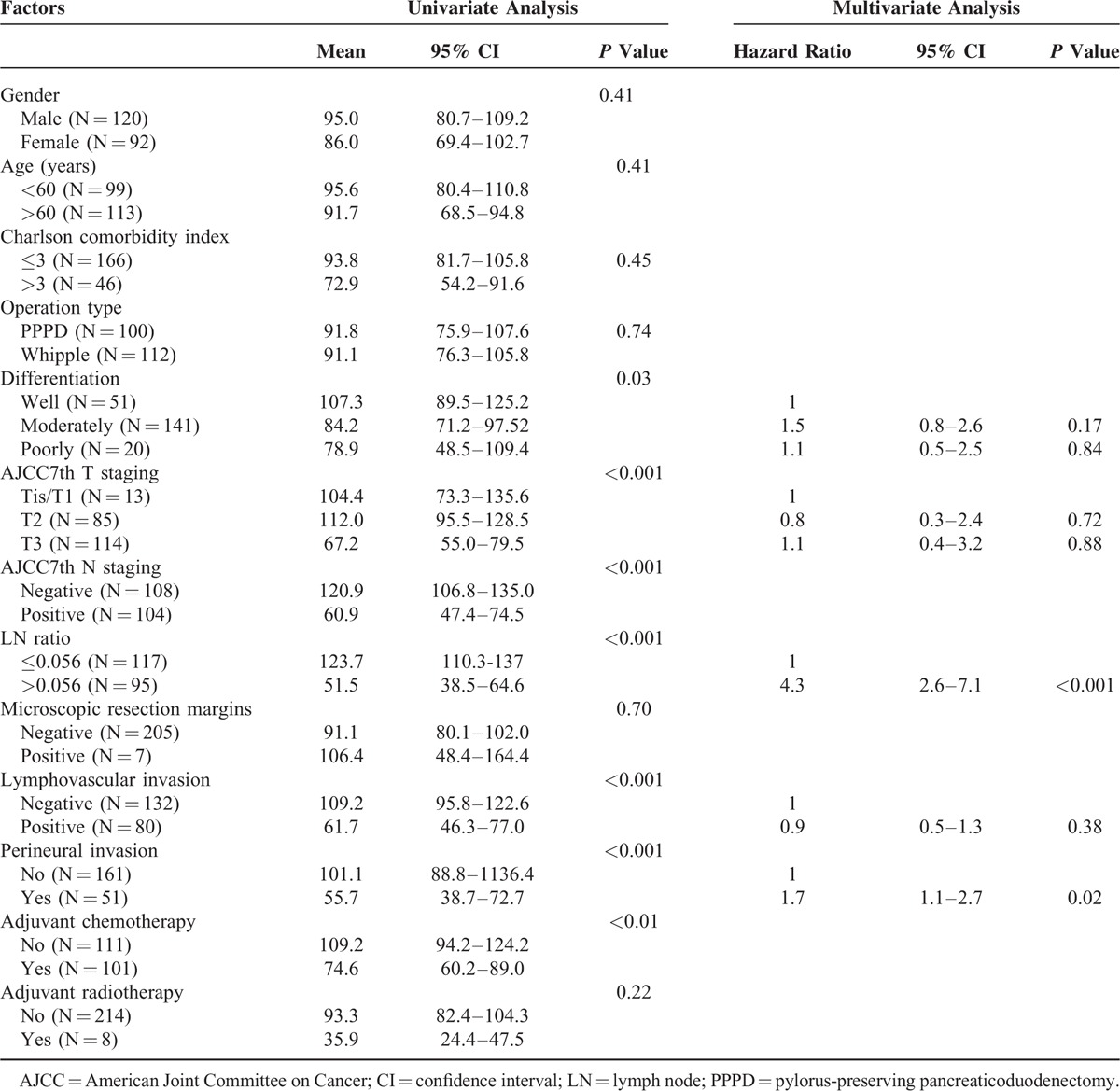
Univariate and Multivariate Analyses of Risk Factors Associated With Overall Survival

**FIGURE 2:**
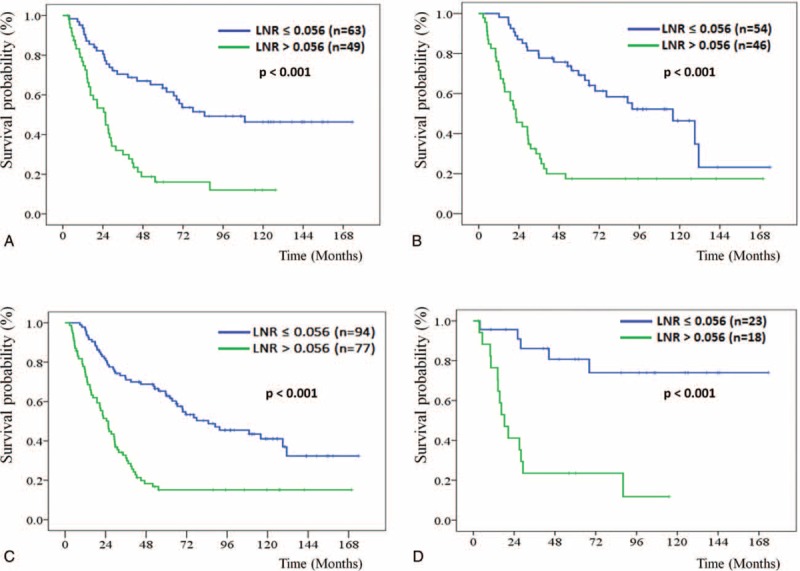
Kaplan–Meier overall survival curves for patients stratified with LNR ≤ 0.056 and >0.056 groups according to the operation method with pancreaticoduodenectomy (A) and pylorus-preserving pancreaticoduodenectomy, PPPD (B), as well as the total number of lymph node resections ≤ 20 (C) and >20 groups (D). LNR = lymph node ratio; PPPD = pylorus-preserving pancreaticoduodenectomy

Patients who underwent PD or PPPD were further stratified by LNR ≤ 0.056 vs >0.056 for OS analysis, respectively. The median OS of the patients with LNR ≤ 0.056 and >0.056 was 84.9 vs 25.5 months (*P* <0.001) for patients who underwent PD (Fig. 2A) and was 115.9 vs 22.2 months (*P* <0.001) for patients who underwent PPPD (Fig. 2B), respectively. Regarding classification by the total number of lymph node resections, the median OS of the patients with LNR ≤ 0.056 and >0.056 was 84.9 vs 25.5 months (*P* <0.001) for the patients with lymph node resection number ≤ 20 group (Fig. 2C) but did not reach 18.3 months (*P* <0.001) for the patients with lymph node resection number >20 group (Fig. 2D), respectively.

### CART Analysis

**FIGURE 3:**
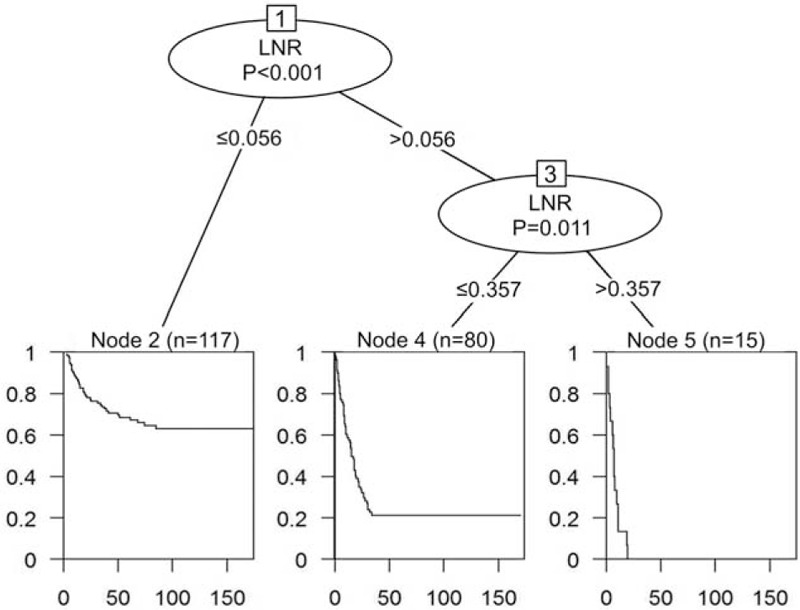
CART analysis identified meaningful prognostic subgroups of metastatic lymph node ratio (LNR) for disease-free survival. The Kaplan–Meier survival curve of each subgroup is presented below each terminal node. CART = classification and regression trees; LNR = lymph node ratio

**FIGURE 4:**
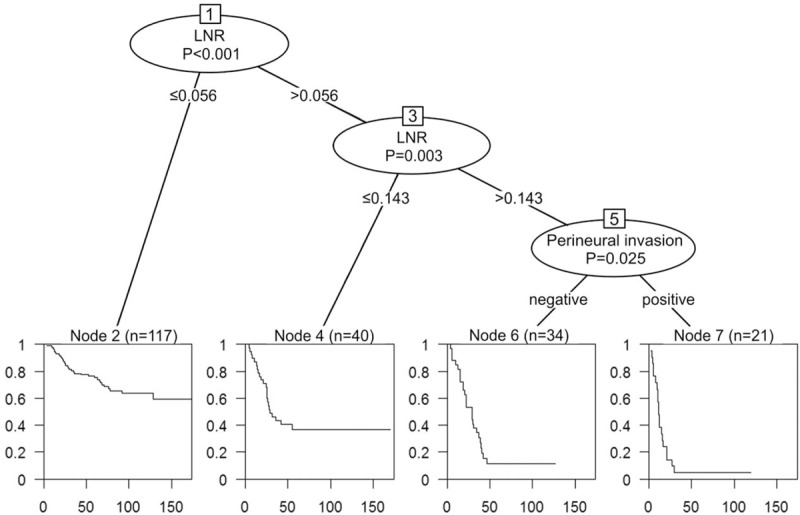
CART analysis for overall survival identified meaningful prognostic subgroups derived from the metastatic lymph node ratio (LNR) and perineural invasion. The Kaplan–Meier survival curve of each subgroup is presented below each terminal node. CART = classification and regression trees; LNR = lymph node ratio.

CART analysis was used to select the most significant cutoff values of LNR for DFS and OS, and the first cutoff value of metastatic LNR for DFS was 0.056. The subgroup with LNR >0.056 was further segregated when LNR was 0.357. These 3 groups of patients had significant difference in DFS outcome (Fig. 3). CART analysis for the prognostic factors of OS also identified that the first cutoff value of metastatic LNR was 0.056. In the subgroup with LNR >0.056, patients with LNR >0.143 had worse OS outcomes than those with LNR between 0.056 and 0.143. Furthermore, status of perineural invasion could divide patients with LNR>0.143 into 2 subgroups with different OS outcomes. The tree algorithm from CART analysis for OS revealed that LNR should be assessed first followed by perineural invasion to predict OS (Fig. 4).

## DISCUSSION

In this study, we present a large series of 212 CAV patients who received surgical treatment (PD and PPPD with regional lymphadenectomy) at a single institution in Asia and report long-term follow-up results (2000–2010). The 5-year OS rate for the CAV patients was 51.1%, and the in-hospital mortality rate was 7.4%. Metastatic LNR >0.056 and perineural invasion were independent poor prognostic factors for OS.

Among the malignancies of the ampulla-pancreatobiliary tract, CAV is rare, with an incidence of 0.49 per 100,000 persons,^18^ but with a better survival rate after surgical treatment.^2,14,19^ Many studies have proposed different prognostic factors and variable outcomes after surgery,^2,4,20–27^ but most of these studies had relatively small patient numbers and were performed at a single institute. We studied a large series, and our results are compatible with those of other reported large series.^5,20,21,23,26,28–31^

Lymph node metastasis has been proposed as a major negative prognosis factor for CAVs^8–12,22,26,27,29,30,32–34^ because it is associated with postoperative liver metastasis and poor OS. The importance of other characteristics of regional lymph node for CAV prognosis, such as metastatic LNR and LNN, is unclear. Hurtuk et al^10^ were the first to review LNR and survival in patients with periampullary malignancies. In their series, 75 CAV patients were grouped according to positive LNR as follows: LNR = 0, LNR≤0.2, LNR≤0.4, and LNR>0.4. Patients in the higher positive LNR group had a significantly poorer prognosis. However, other studies^11,12,14^ have revealed that metastatic LNN would predict a worse prognosis than metastatic LNR in multivariate analysis. Most of these studies did not explain how the optimal cutoff value of LNN and LNR in CAV was determined. In our review, we used CART analysis rather than the Cox proportional hazards model to determine meaningful prognostic subgroups for the continuous prognostic factors such as LNN and LNR. Based on CART analysis, the optimal cutoff value of metastatic LNR was 0.056. In our study, LNR >0.056 was a strong negative prognostic factor for both DFS and OS in the multivariate analysis. Concerning the influence of LNR value among patients with a lower total number of lymph node resections and different operation types (PD or PPPD), patients who underwent PD or PPPD and had lymph node resection number ≤ 20 and >20 were further stratified by LNR ≤ 0.056 vs >0.056 for overall survival (OS) and disease-free survival (DFS) analyses. Our results showed LNR ≤ 0.056 vs >0.056 significantly discriminated both OS and DFS, regardless of operation type or total number of lymph node resections. To our knowledge, this is the first study to use CART analysis to determine the optimal cutoff value of LNR in CAV patients.

Several pathologic characteristics, including histological differentiation, tumor's gross appearance, vascular invasion, lymphatic invasion, and perineural invasion,^5,7,12,20,22,27,29,35–39^ have been reported to be associated with prognosis of CAV. However, only a few series have discussed perineural invasion as a prognostic factor for CAV.^5,12,13,30,40–44^ Duffy et al^42^ reported a 55-patient series, with 21% of patients having perineural invasion. They found that perineural invasion was a significantly poorer prognostic factor compared to the lymph node status (HR = 20.151 vs HR = 0.971; *P* <0.001 vs *P* = 0.98). Nakai et al^43^ studied a 25-patient series and found microperineural invasion by the antinerve fiber antibody (S-100) in the resected CAV specimens. In the multivariate analysis, microperineural invasion was found to be the most important prognostic factor for CAV. Our series revealed that perineural invasion (24%) was a poor prognostic factor for OS, but its impact was less significant than LNR in predicting OS by CART analysis.

There are some limitations to our study. Given the study's retrospective nature and the long study period, some of our patients did not receive regular follow-up in our hospital. Therefore, we were not able to analyze the actual survival outcomes after surgical treatment at our institute. Different surgeons with varying ability performed the operation for these patients, and the treatment strategies might have changed over time, which is beyond our control, likely contributing to different therapeutic outcomes. Finally, some of the patients had been received adjuvant chemotherapy or/and radiotherapy, as such there was selection bias regarding which patients were offered the adjuvant treatment. The effectiveness of adjuvant therapy may also potentially affect patient's outcome. Further large studies are needed to verify our results in the future.

In conclusion, we have reported a large series of 212 CAV patients at a single institution in Asia and provided long-term follow-up results. A PPPD or traditional PD can be performed on CAV patients with a low surgical mortality rate and an acceptable 5-year OS rate (51.1%). Perineural invasion in patients with resected CAV was also associated with poor OS. Moreover, a metastatic LNR >0.056 predicted both a high tumor recurrence rate and a poor OS rate. Greater awareness of the impact of metastatic LNR may help clinicians provide appropriate adjuvant treatment for high-risk CAV patients after curative surgery.
